# Parkinson’s disease: animal models and dopaminergic cell vulnerability

**DOI:** 10.3389/fnana.2014.00155

**Published:** 2014-12-15

**Authors:** Javier Blesa, Serge Przedborski

**Affiliations:** Department of Pathology and Cell Biology, Center for Motor Neuron Biology and Disease, College of Physicians and Surgeons, Columbia UniversityNew York, NY, USA

**Keywords:** MPTP, 6-OHDA, rotenone, synuclein, LRRK2, parkin, DJ1, ATP13A2

## Abstract

Parkinson’s disease (PD) is a neurodegenerative disorder that affects about 1.5% of the global population over 65 years of age. A hallmark feature of PD is the degeneration of the dopamine (DA) neurons in the substantia nigra pars compacta (SNc) and the consequent striatal DA deficiency. Yet, the pathogenesis of PD remains unclear. Despite tremendous growth in recent years in our knowledge of the molecular basis of PD and the molecular pathways of cell death, important questions remain, such as: (1) why are SNc cells especially vulnerable; (2) which mechanisms underlie progressive SNc cell loss; and (3) what do Lewy bodies or α-synuclein reveal about disease progression. Understanding the variable vulnerability of the dopaminergic neurons from the midbrain and the mechanisms whereby pathology becomes widespread are some of the primary objectives of research in PD. Animal models are the best tools to study the pathogenesis of PD. The identification of PD-related genes has led to the development of genetic PD models as an alternative to the classical toxin-based ones, but does the dopaminergic neuronal loss in actual animal models adequately recapitulate that of the human disease? The selection of a particular animal model is very important for the specific goals of the different experiments. In this review, we provide a summary of our current knowledge about the different *in vivo* models of PD that are used in relation to the vulnerability of the dopaminergic neurons in the midbrain in the pathogenesis of PD.

## INTRODUCTION

Parkinson’s disease (PD) is a common neurodegenerative disorder whose prevalence increases with age (Pringsheim et al., 2014). The cardinal features of PD include tremor, rigidity and slowness of movements, albeit non-motor manifestations such as depression and sleep disturbances are increasingly recognized in these patients (Rodriguez-Oroz et al., 2009). Over the past decade, more attention has also been paid to the broader nature of the neurodegenerative changes in the brains of PD patients. Indeed, for many years, the neuropathological focus has been on the striking neurodegeneration of the nigrostriatal dopaminergic pathway, however, nowadays, disturbances of the serotonergic, noradrenergic, glutamatergic, GABAergic, and cholinergic systems (Brichta et al., 2013) as well as alterations in neural circuits are now being intensively investigated from the angle of the pathophysiology of PD (Obeso et al., 2014), with the underlying expectation of acquiring a better understanding of the neurobiology of this disabling disorder and of identifying new targets for therapeutic purposes. From a molecular biology point of view, the accepted opinion that the PD neurodegenerative process affects much more than the dopaminergic neurons of the substantia nigra pars compacta (SNc), has triggered a set of fascinating questions such as: are dopaminergic and non-dopaminergic neurons in PD dying by the same pathogenic mechanisms; and, given the fact that within a given subtype of neurons, not all die to the same extent nor at the same rate [e.g., dopaminergic neurons in the SNc vs. ventral tegmental area (VTA)], what are the molecular determinants of susceptibly/and resistance to disease?

To gain insights into these types of critical questions, a brief review of the literature demonstrates that the enthusiasm for experimental models of PD, both *in vitro* and *in vivo*, has greatly increased, in part, thanks to new strategies for producing sophisticated models, such as the temporal- and/or cell-specific expression of mutated genes in mice (Dawson et al., 2010), human pluripotent cells coaxed into a specific type of neurons (Berg et al., 2014), and a host of invertebrate organisms like *Drosophila* (Guo, 2012), *Caenorhabditis elegans* (Chege and McColl, 2014), or Medaka fish (Matsui et al., 2014). Thus far, however, all of these experimental models continue to be categorized into two main flavors: toxic and genetic (and sometimes, both approaches are combined). But, more importantly, none of the currently available models phenocopy PD, mainly because they lack some specific neuropathological and/or behavioral feature of PD. Some PD experts see this as fatal flaws, while others tend to ignore the shortcomings. It has always been our personal view that models are just models and, as such, given the large collection of models the field of PD possesses, the prerequisite resides in not using just any model but in selecting the optimal *in vitro* or *in vivo* model whose strengths are appropriate for investigating the question being asked and whose weaknesses will not invalidate the interpretation of an experiment.

Based on our above premise, herein, we discuss the experimental models of PD, with a deliberate emphasis on *in vivo* mammalian models induced by reproducible means. Over the years, a constellation of uncommon strategies and organisms have been used to produce models of PD. However, in this review, we have decided not to discuss these cases, because we have limited space and because we are missing sufficient independent information to assessment the reproducibility and reliability of these models, which, to us, is critical for distinguishing between interesting “case reports” and useful tools to model human diseases.

## TOXIN MODELS

A number of pharmacological and toxic agents including reserpine, haloperidol, and inflammogens like lipopolysaccharide have been used over the years to model PD, although the two most widely used are still the classical 6-OHDA in rats and MPTP in mice and monkeys. Although the neurotoxic models appear to be the best ones for testing degeneration of the nigrostriatal pathway, some striking departures from PD need to be mentioned: the degeneration of dopaminergic neurons progress rapidly, i.e., days not years, lesions are primarily if not exclusively dopaminergic, and animals lack the typical PD proteinaceous inclusions called Lewy bodies (LBs). In addition, behavioral abnormalities in these animal models are also a challenging question (see below; **Table 1**).

**Table 1:** Animal models of Parkinson disease.

	Animal model	Motor behavior	SNc neuron loss	Striatal DA loss	Lewy body/Syn pathology
Toxin-based	MPTP Mice	Reduced locomotion, bradykinesia	**↑ ↑ ↑**	**↑ ↑ ↑**	NO
	MPTP Monkeys	Reduced locomotion, altered behavior, tremor, and rigidity	**↑ ↑ ↑**	**↑ ↑ ↑**	NO
	6-OHDA rat	Reduced locomotion, altered behavior	**↑ ↑ ↑**	**↑ ↑ ↑**	NO
	Rotenone	Reduced locomotion	**↑ ↑**	**↑ ↑ ↑**	YES
	Paraquat/maneb	Reduced locomotion	**↑ ↑**	**↑ ↑ ↑**	YES
	MET/MDMA	Reduced locomotion	**↑ ↑**	**↑ ↑ ↑**	NO
Genetic mutations*	α-Synuclein	Altered behavior, reduced or increased motor activity	**↑** Not consistent	**↑**	**↑** (in old animals)
	LRKK2	Mild behavioral alteration	NO	NO	NO
	PINK1	No obvious alterations or reduced locomotion	NO	NO	NO
	PARKIN	No obvious locomotion or reduced locomotion	NO	**↑**	NO
	DJ-1	Decreased locomotor activity	NO	NO	NO
	ATP13A2	Late onset sensorimotor deficits	NO	NO	NO
Others	SHH	Reduced locomotion	**↑ ↑**	**↑ ↑**	NO
	Nurr1	Reduced locomotion	**↑ ↑**	**↑ ↑**	NO
	Engrailed 1	Reduced locomotion	**↑ ↑**	**↑**	NO
	Pitx3	Reduced locomotion	**↑ ↑ ↑**	**↑ ↑ ↑**	NO
	C-Rel-NFKB	Gait, bradykinesia, rigidity	**↑ ↑**	**↑ ↑**	YES
	MitoPark	Reduced locomotion, tremor, and rigidity	**↑ ↑**	**↑ ↑**	YES
	Atg7	Late onset locomotor deficits	**↑ ↑**	**↑ ↑**	YES
	VMAT2	Reduced locomotion and altered behavior	**↑ ↑**	**↑ ↑**	YES

*↑↑↑, Severe loss; ↑↑, Moderate loss; ↑, Mild loss*.

*This table summarizes general observations for each model. See the main text for full and specific description of different animal models for each genetic mutation.

### MPTP

MPTP is the tool of choice for investigations into the mechanisms involved in the death of DA neurons in PD. MPTP has been shown to be toxic in a large range of species (Tieu, 2011). The most popular species, besides primates, is the mouse, as rats were found to be resistant to this toxin (Chiueh et al., 1984). A number of intoxication regimens or administration methods have been used over the years in mouse (Jackson-Lewis and Przedborski, 2007; Meredith et al., 2008) and in primates (Bezard et al., 1997; Blesa et al., 2012; Porras et al., 2012). In both species, MPTP primarily causes damage to the nigrostriatal DA pathway with a profound loss of DA in the striatum and SNc (Dauer and Przedborski, 2003).

This specific and reproducible neurotoxic effect on the nigrostriatal system is the strength of this model. Neuropathological data show that MPTP administration causes damage to the nigrostriatal DA pathway that is identical to that seen in PD (Langston et al., 1983), yet there is a resemblance that goes beyond the loss of SNc DA neurons. Like in PD, MPTP causes greater loss of DA neurons in SNc than in VTA or retrorubral field (Seniuk et al., 1990; Muthane et al., 1994; Blesa et al., 2011, 2012) and, at least in monkeys treated with low doses of MPTP, greater degeneration of DA nerve terminals in the putamen than in the caudate nucleus (Moratalla et al., 1992; Snow et al., 2000; Blesa et al., 2010).

A often raised weakness with this model is the lack of LB (Shimoji et al., 2005; Halliday et al., 2009). Although no LBs have been observed in these models so far, a few reports have investigated the expression, regulation or pattern of α-syn after MPTP exposure (Vila et al., 2000; Dauer et al., 2002; Purisai et al., 2005). Only, in MPTP-injected monkeys, have intraneuronal inclusions, reminiscent of LBs, been described (Forno et al., 1986; Kowall et al., 2000). Behavior is also an issue, and except for the monkeys, features reminiscent of PD are lacking especially in mice. Yet, using a battery of tests, some motor alterations in mice with profound dopaminergic deficit may be detected (Taylor et al., 2010).

### 6-OHDA

Like MPTP, 6-OHDA is a selective catecholaminergic neurotoxin that is used, mainly, to generate lesions in the nigrostriatal DA neurons in rats (Ungerstedt, 1968). Since 6-OHDA cannot cross the blood-brain barrier, systemic administration fails to induce parkinsonism. So, this induction model requires that 6-OHDA be injected (typically as a unilateral injection) into the SNc, medial forebrain bundle or striatum (Blandini et al., 2008). Intraventricular administration has also been achieved (Rodríguez Díaz et al., 2001). The effects resemble those in the acute MPTP model, causing neuronal death over a brief time course (12 h to 2–3 days). The intrastriatal injection of 6-OHDA causes progressive retrograde neuronal degeneration in the SNc and VTA (Sauer and Oertel, 1994; Przedborski et al., 1995). The pattern of DA loss in animals bearing a full lesion (>90%) again mirrors seen that in PD, with the SNc showing more cells loss compared to the VTA (Przedborski et al., 1995). As in PD, DA neurons are killed, and the non-DA neurons are preserved. However, like in the MPTP model, 6-OHDA does not produce LB-like inclusions in the nigrostriatal pathway. Traditionally, behavioral assessments of motor impairments in the unilateral 6-OHDA model are done by drug-induced rotation tests (Dunnett and Lelos, 2010). However, drug-free sensorimotor behavioral tests have been developed in both rat and mice that may be helpful for the preclinical testing of new symptomatic strategies (Schallert et al., 2000; Glajch et al., 2012).

### ROTENONE

Chronic systemic exposure to rotenone in rats causes many features of PD, including nigrostriatal DA degeneration (Betarbet et al., 2000). The rotenone-administered animal model also reproduces all of the behavioral features reminiscent of human PD. Importantly, many of the degenerating neurons have intracellular inclusions that resemble LB morphologically. These inclusions show immunoreactivity for α-syn and ubiquitin as did the original LB (Sherer et al., 2003). Usually, rotenone is administered by daily intraperitoneal injection (Cannon et al., 2009), intravenously or subcutaneously (Fleming et al., 2004). Recently, rotenone has been tested in mice through chronic intragastric administration, (Pan-Montojo et al., 2010) or as a stereotaxic injection or infusion directly in the brain (Alam et al., 2004; Xiong et al., 2009) recapitulating the slow and specific loss of DA neurons. However, administration of rotenone in rats causes high mortality and, somehow, is difficult to replicate.

### PARAQUAT/MANEB

Although the idea that the herbicide paraquat (N,N′-dimethyl-4-4-4′-bypiridinium), may cause parkinsonism in humans has attracted some interest, at this time, as pointed out by Berry and collaborators, epidemiological and clinical evidence that paraquat may cause PD is inconclusive (Berry et al., 2010). And, the same view seems to apply to the fungicide maneb (manganese ethylenebisdithiocarbamate; Berry et al., 2010). Moreover, effects of this compound in the nigrostriatal DA system is somewhat ambiguous (Freire and Koifman, 2012). Regarding animal models, some researchers report that, following the systemic application of paraquat, mice exhibit reduced motor activity and a dose-dependent loss of striatal tyrosine hydroxylase (TH) fibers and SNc neurons with relative sparing of the VTA (Brooks et al., 1999; Day et al., 1999; McCormack et al., 2002; Rappold et al., 2011). Like rotenone, paraquat may be useful in the laboratory because of its presumed ability to induce LB in DA neurons (Manning-Bog et al., 2002). Maneb has been shown to decrease locomotor activity and produce SNc neurons loss (Thiruchelvam et al., 2003) and potentiate both the MPTP and the paraquat effects (Takahashi et al., 1989; Thiruchelvam et al., 2000; Bastías-Candia et al., 2013). However, as with rotenone, this model shows contradictory results, variable cell death and loss of striatal DA content (Miller, 2007).

### AMPHETAMINE-TYPE PSYCHOSTIMULANTS

Some amphetamine derivatives such as methamphetamine (METH) and 3,4-methylenedioxymethamphetamine (MDMA) also have neurotoxic effects on the nervous system causing not only functional deficits but also structural alterations (Cadet et al., 2007; Thrash et al., 2009). The first study to show DA depletion in rats following repeated, high-dose exposure to METH was conducted by Kogan et al. (1976). Hess et al. (1990) and Sonsalla et al. (1996) showed that high-dose treatment with METH in mice resulted in a loss of DA cells in the SNc. Since then, several studies have reported selective DA or serotonergic nerve terminal as well as SNc neuronal loss in rodents, primates or even guinea pig following the administration of very high doses of METH (Wagner et al., 1979; Trulson et al., 1985; Howard et al., 2011; Morrow et al., 2011).

3,4-Methylenedioxymethamphetamine can also elicit significant neurobehavioral adverse effects. Although MDMA toxicity mainly affects the serotonergic system, DA system can also be affected to a lesser extent (Jensen et al., 1993; Capela et al., 2009). In mice, repeated administration of MDMA produces degeneration of DA terminals in the striatum (O’Callaghan and Miller, 1994; Granado et al., 2008a,b) and TH+ neuronal loss in the SNc (Granado et al., 2008b).

Exposure to low concentrations of METH results in a decrease of the vulnerability of the SNc DA cells to toxins like 6-OHDA or MPTP (Sziráki et al., 1994; El Ayadi and Zigmond, 2011). On the other hand, chronic exposure to MDMA of adolescent mice exacerbates DA neurotoxicity elicited by MPTP in the SNc and striatum at adulthood (Costa et al., 2013). Hence, a METH or MDMA-treated animal model could be useful to study the mechanisms of DA neurodegeneration (Thrash et al., 2009).

## GENETIC MODELS

Genetic models may better simulate the mechanisms underlying the genetic forms of PD, even though their pathological and behavioral phenotypes are often quite different from the human condition. A number of cellular and molecular dysfunctions have been shown to result from these gene defects like fragmented and dysfunctional mitochondria (Exner et al., 2012; Matsui et al., 2014; Morais et al., 2014), altered mitophagy (Lachenmayer and Yue, 2012; Zhang et al., 2014), ubiquitin–proteasome dysfunction (Dantuma and Bott, 2014), and altered reactive oxygen species production and calcium handling (Gandhi et al., 2009; Joselin et al., 2012; Ottolini et al., 2013). Some studies have reported alterations in motor function and behavior in these mice (Hinkle et al., 2012; Hennis et al., 2013; Vincow et al., 2013), and sensitivities to complex I toxins, like MPTP, different from wild type (WT) mice (Dauer et al., 2002; Nieto et al., 2006; Haque et al., 2012) although this latter finding is not always consistent (Rathke-Hartlieb et al., 2001; Dong et al., 2002). However, almost all of the studies evaluating the integrity of the nigrostriatal DA system in these genetic models failed to find significant loss of DA neurons (Goldberg et al., 2003; Andres-Mateos et al., 2007; Hinkle et al., 2012; Sanchez et al., 2014). Thus, recapitulation of the genetic alterations in mice is insufficient to reproduce the final neuropathological feature of PD. Below, we describe transgenic mice or rat models which recapitulate the most known mutations observed in familial PD patients (**Table 1**).

### α-SYNUCLEIN

α-syn was the first gene linked to a dominant-type, familial PD, called Park1, and is the main component of LB which are observed in the PD brain (Goedert et al., 2013). Three missense mutations of α-syn, encoding the substitutions A30P, A53T, and E46K, have been identified in familial PD so far (Vekrellis et al., 2011; Schapira et al., 2014). Furthermore, the duplication or triplication of α-syn is sufficient to cause PD, suggesting that the level of α-syn expression is a critical determinant of PD progression (Singleton et al., 2003; Kara et al., 2014).

To date, various α-syn transgenic mice have been developed. Although, in some of these mice, decreased striatal levels of TH or DA and behavioral impairments indicate that the accumulation of α-syn can significantly alter the functioning of DA neurons, no significant nigrostriatal degeneration has been found in most of them. The models of α-syn overexpression in mice recapitulate the neurodegeneration, depending primarily on the promoter used to drive the expression of the transgene, whether the transgene codes for the WT or the mutated protein, and the level of expression.

Although a lot of behavioral alterations have been described in both the A30P and A53T mice (Sotiriou et al., 2010; Oaks et al., 2013; Paumier et al., 2013), the mouse prion protein promoter failed to reproduce the cell loss in the SNc or locus coeruleus (LC; van der Putten et al., 2000; Giasson et al., 2002; Gispert et al., 2003). The same phenotype was found with the hamster prion promoter (Gomez-Isla et al., 2003). Mice based on the PDGF-β promoter showed loss of terminals and DA in the striatum but no TH+ cell loss (Masliah et al., 2000). The TH promoter led to TH+ cell loss only in a few studies (Thiruchelvam et al., 2004; Wakamatsu et al., 2008) but did not replicate the α-syn neuropathology as did the Thy-1 promoter (Matsuoka et al., 2001; Chen et al., 2006; Miller et al., 2007; Su et al., 2009). However, the use of the murine Thy-1 promoter often causes loss of DA levels in the striatum but only moderate nigral DA cell loss in the SNc, with α-syn pathology (van der Putten et al., 2000; Rockenstein et al., 2002; Ikeda et al., 2009; Ono et al., 2009; Lam et al., 2011). A new line of tetracycline-regulated inducible transgenic mice that overexpressed α-syn A53T under control of the promoter of Pitx3 in the DA neurons developed profound motor disabilities and robust midbrain neurons neurodegeneration, profound decrease of DA release, the fragmentation of Golgi apparatus, and the impairments of autophagy/lysosome degradation pathways (Lin et al., 2012). Janezic et al. (2013) generated BAC transgenic mice (SNCA-OVX) that express WT human α-syn and which display an age-dependent loss of SNc DA neurons preceded by early deficits in DA release from terminals in the dorsal striatum, protein aggregation and reduced firing of SNc DA neurons. Regarding the transgene expressed, the A53T seems to be more effective than the A30P, in general.

Several viral vectors, primarily lentiviruses and adeno-associated viruses (AAVs), have been used to drive exogenous α-syn. Rats are usually used for these studies because viral vector delivery requires stereotactic injections within or near the site of the neuronal cell bodies in the SNc (Kirik et al., 2002; Klein et al., 2002; Lo Bianco et al., 2002; Lauwers et al., 2003, 2007). In contrast to all of the α-syn transgenic mice, viral vector-mediated α-syn models display α-syn pathology and clear dopaminergic neurodegeneration. The injection of human WT or A53T mutant α-syn by AAVs into the SNc neurons of rats induces a progressive, age-dependent loss of DA neurons, motor impairment, and α-syn cytoplasmic inclusions (Kirik et al., 2002; Klein et al., 2002; Lo Bianco et al., 2002; Decressac et al., 2012). This cell loss was preceded by degenerative changes in striatal axons and terminals, and the presence of α-syn positive inclusions in axons and dendrites (Kirik et al., 2003; Decressac et al., 2012). These results have been replicated in mice (Lauwers et al., 2003; Oliveras-Salvá et al., 2013). Although these models still suffer from a certain degree of variability, they can be of great value for further development and testing of neuroprotective strategies.

Recently, several studies have demonstrated that α-syn may be transmissible from cell to cell (Luk and Lee, 2014). In WT mice, a single intrastriatal inoculation of synthetic α-syn fibrils or pathological α-syn purified from postmortem PD brains led to the cell-to-cell transmission of pathologic α-syn and LB pathology in anatomically interconnected regions and was accompanied by a progressive loss of dopaminergic neurons in the SNc and reduced DA levels in the striatum, culminating in motor deficits (Luk et al., 2012a,b; Masuda-Suzukake et al., 2014; Recasens et al., 2014). Moreover, the hind limb intramuscular injection of α-syn can induce pathology in the central nervous system in transgenic mouse models (Sacino et al., 2014).

### LRKK2

Mutations in LRRK2 are known to cause a late-onset autosomal dominant inherited form of PD (Healy et al., 2008). Several mutations have been identified in LRRK2, the most frequent being the G2019S mutation, a point mutation in the kinase domain, whereas R1441C, a mutation in the guanosine triphosphatase domain, is the second most common (Rudenko and Cookson, 2014). Overall, LRRK2 mice models display mild or not functional disruption of the nigrostriatal DA neurons of the SNc.

LRRK2 KO mice are viable and have an intact nigrostriatal DA pathway up to 2 years of age. Neuropathological features associated with neurodegeneration or altered neuronal structure were absent, but α-syn or ubiquitin accumulation has been reported in these mice (Andres-Mateos et al., 2009; Lin et al., 2009; Tong et al., 2010; Hinkle et al., 2012). To date, two LRRK2 KO rat models have been developed, although the consequences of LRRK2 deficiency in the brain are still unknown (Baptista et al., 2013; Ness et al., 2013).

Both G2019S and R1441C LRRK2 KI mice are viable, fertile, and appear grossly normal. This mutation had no impact on DA neuron number or morphology in the SNc, or on noradrenergic neurons in the LC. Striatal DA levels and DA turnover are also normal in these mice (Tong et al., 2009; Herzig et al., 2011).

Overexpression of G2019S LRRK2 leads to a mild progressive and selective degeneration of SNc DA neurons (20%) up to 2 years of age. Furthermore, no alteration in striatal DA levels or locomotor activity could be detected in older G2019S LRRK2 mice (Ramonet et al., 2011; Chen et al., 2012). Also, Maekawa et al. (2012) generated transgenic mice constitutively expressing V5-tagged human I2020T LRRK2 from a CMV promoter with no influence on SNc DA neuronal number or striatal DA fiber density. Zhou et al. (2011) developed a transgenic rat model expressing G2019S LRRK2. Despite a mild behavioral alteration, LRRK2 expression had no effect on the number of DA neurons or on striatal DA content. Recently, conditional expression of R1441C LRRK2 in midbrain dopaminergic neurons of mice results in nuclear abnormalities but, without neurodegeneration (Tsika et al., 2014).

Additional LRRK2 BAC transgenic mouse models have also been developed. These mice displayed age-dependent and progressive motor deficits at 10–12 months of age, accompanied by a mild reduction of striatal DA release. Adult neurogenesis and neurite outgrowth are impaired. No DA neurons loss or degeneration of striatal nerve terminals where observed in mice at 9–10 months of age (Li et al., 2009b, 2010; Melrose et al., 2010; Winner et al., 2011).

Regarding the viral vector-based models, Lee et al. (2010) developed a herpes simplex virus (HSV) amplicon-based mouse model of G2019S LRRK2-induced DA neurotoxicity. The nigrostriatal expression of WT LRRK2 induced modest nigral DA neurodegeneration (10–20%), whereas expression of the kinase-hyperactive G2019S LRRK2 resulted in a 50% neuronal loss in the ipsilateral SNc associated with reduced striatal DA fiber density at 3 weeks post-injection. In another study, a model based on the unilateral injection of recombinant, second-generation human serotype 5 adenoviral (rAd) vectors expressing FLAG-tagged human WT or G2019S LRRK2 driven by a neuronal-specific human synapsin-1 promoter in rats induced the progressive loss (20%) of DA neurons in the ipsilateral SNc over 42 days, but with no reduction of striatal DA fiber density (Dusonchet et al., 2011).

### PINK1

Mutations in the gene PINK1 cause another form of PD called PARK6 (Scarffe et al., 2014). PINK1 KO mice have an age-dependent, moderate reduction in striatal DA levels accompanied by low locomotor activity, but do not exhibit major abnormalities in the DA neurons or striatal DA levels (Gautier et al., 2008; Gispert et al., 2009). These mice showed no LB formation or nigrostriatal degeneration for up to 18 months of age. However, in PINK1 KO mice, overexpression of α-syn in the SNc resulted in enhanced dopaminergic neuron degeneration as well as significantly higher levels of α-syn phosphorylation at serine 129 at 4 weeks post-injection (Oliveras-Salvá et al., 2014). Recently, a PINK1 null mouse with an exon 4–5 deletion displayed a progressive loss of DA in the striatum, but there was no degeneration in the SNc (Akundi et al., 2011). The phenotypes of these mice are very similar to those of Parkin KO and DJ-1 KO mice.

### PARKIN

Parkin is an E3 ubiquitin ligase that functions in the ubiquitin–proteasome system. Mutations in parkin are a cause of familial PD and are also seen in some young-onset sporadic PD cases (Lücking et al., 2000; Periquet et al., 2003). Several parkin KO mice have been generated, typically produced by deletion at exon 3, exon 7, or exon 2 in the PRKN gene (Goldberg et al., 2003; Itier et al., 2003; Palacino et al., 2004; Von Coelln et al., 2004; Perez and Palmiter, 2005; Zhu et al., 2007; Martella et al., 2009). However, they show no substantial DA-related behavioral abnormalities. Some of these KO mice exhibit slightly impaired DA release (Itier et al., 2003; Kitada et al., 2009a) and reduced norepinephrine levels in the olfactory bulb and spinal cord with an abnormal nigrostriatal region but without loss of SNc neurons (Goldberg et al., 2003; Von Coelln et al., 2004).

Only the Parkin-Q311X-DAT-BAC mice exhibit multiple late onsets and progressive hypokinetic motor deficits, age-dependent DA neuron degeneration in the SNc and a significant reduction in striatal DA and dopaminergic terminals in the striatum (Lu et al., 2009). Recently, overexpression of T240R-parkin and of human WT parkin induced progressive and dose-dependent DA cell death in rats (Van Rompuy et al., 2014).

### DJ-1

DJ-1 mutations are linked to an autosomal recessive, early onset PD (Puschmann, 2013). KO models of DJ-1 mice with a targeted deletion of exon 2 or insertion of a premature stop codon in exon 1 show decreased locomotor activity, a reduction in the release of evoked DA in the striatum but no loss of SNc DA neurons and no change of the DA levels (Goldberg et al., 2005; Kim et al., 2005). However, one line of DJ-1 KO mice shows loss of DA neurons in the VTA (Pham et al., 2010).

Interestingly, a recently described DJ-1 KO mouse, backcrossed on a C57/BL6 background, displayed a dramatic early onset unilateral loss of DA neurons in the SNc, progressing to bilateral degeneration of the nigrostriatal axis, with aging. In addition, these mice exhibit age-dependent bilateral degeneration in the LC and display, with aging, a mild motor behavioral deficit at specific time points (Rousseaux et al., 2012). Therefore, if confirmed, this new mouse model would provide a tool to study the preclinical aspects of PD.

### ATP13A2

Mutations in ATP13A2 (PARK9), encoding a lysosomal P-type ATPase, are associated with both Kufor–Rakeb syndrome (KRS) and neuronal ceroid lipofuscinosis. KRS has recently been classified as a rare genetic form of PD (Heinzen et al., 2014; Yang and Xu, 2014). Despite the accumulation of lipofuscin deposits in the SNc and late-onset sensorimotor deficits, there was no change in the number of DA neurons in the SNc or in striatal DA levels in aged Atp13a2 KO mice (Schultheis et al., 2013).

## OTHER MODELS

Inactivation of multiple PD genes has been shown to be insufficient to cause significant nigral degeneration within the lifespan of mice (Hennis et al., 2014). Triple KO mice lacking Parkin, DJ-1, or PINK1 have normal morphology and normal numbers of dopaminergic and noradrenergic neurons in the SNc and LC. Also, levels of striatal DA in these triple KO mice were normal at 16 months, but increased at 24 months of age (Kitada et al., 2009b).

Sonic hedgehog (SHH), nuclear receptor related protein-1 (Nurr1), pituitary homeobox3 (Pitx3), and engrailed 1 (EN1) are transcription factors important to the development and maintenance of the nigro-striatal system (Jankovic et al., 2005; Jiang et al., 2005; Li et al., 2009a; Gonzalez-Reyes et al., 2012; Zhang et al., 2012). Both SHH and Nurr1 KO mice show a progressive loss of DA neurons without LB formation (Jiang et al., 2005; Kadkhodaei et al., 2009; Gonzalez-Reyes et al., 2012). Also, Pitx3 gene mutations cause a complete loss of SNc and VTA DA neurons and altered locomotor activity in mice (Hwang et al., 2003; van den Munckhof et al., 2003). Recently, engrailed 1 heterozygous mice (En1+/–) showed a significant and progressive retrograde degeneration of SNc neurons and dystrophic and swollen striatal TH+ terminals (Nordström et al., 2014). c-Rel (a subunit of the NFκB complex) KO mice also develop a PD-like neuropathology on aging. At 18 months of age, c-rel (–/–) mice exhibit a significant loss of DA neurons in the SNc, loss of dopaminergic terminals and a significant reduction of DA and HVA levels in the striatum. In addition, these mice show age-dependent deficits in locomotor activity and a marked immunoreactivity for fibrillary α-syn in the SNc (Baiguera et al., 2012).

Conditional disruption of the gene for mitochondrial transcription factor A in DA neurons (MitoPark) results in a parkinsonism phenotype in mice that includes an adult-onset, slowly progressive impairment of motor function, DA neuron death, degeneration of nigrostriatal pathways and intraneuronal inclusions (Ekstrand et al., 2007; Good et al., 2011). Also, cell-specific deletion of the essential autophagy gene Atg7 in midbrain DA neurons causes DA neuron loss in the SNc at 9 months, accompanied by late-onset locomotor deficits. Atg7-deficient DA neurons in the midbrain also exhibit early dendritic and axonal dystrophy, reduced striatal DA content, and the formation of somatic and dendritic ubiquitinated inclusions (Friedman et al., 2012).

Recently, it has been suggested that a vesicular monoamine tranporter (VMAT2) defect may be an early abnormality promoting mechanisms leading to nigrostriatal DA neuron death in PD (Pifl et al., 2014). VMAT2-deficient mice display a progressive loss of nigral DA and LC cells, loss of striatal DA and α-syn accumulation (Taylor et al., 2011, 2014). Neuroprotection from MPTP toxicity in VMAT2-overexpressors and enhanced MPTP toxicity in VMAT2-KO mice suggest that interventions aimed at enhancing vesicular capacity may be of therapeutic benefit in PD (Takahashi et al., 1997; Lohr et al., 2014).

## CONCLUDING REMARKS

Despite the significant contribution of all of these animal models to our understanding of PD, none of these models reproduce the human condition. If we consider toxic models, significant nigrostriatal degeneration is generally obtained with some motor deficits (particularly in MPTP-treated monkeys). Although no consistent LB-like formation is detected, this issue in the study of PD pathogenesis remains to be demonstrated. On the other hand, although transgenic models offer insights into the causes of PD pathogenesis or LB-like formation, the absence of consistent neuronal loss in the SNc remains a major limitation for these models. Another troubling observation in genetic models is the often inconsistent phenotypes among the lines with the same mutations. Whether or not this is related to an artifact of insertion of the transgene or to the actual genetic background, it would be advisable to test these in more than one line.

In addition to the classical motor abnormalities observed in PD, animal models are increasingly used to study non-motor symptoms (sleep disturbances, neuropsychiatric and cognitive deficits; Campos et al., 2013; Drui et al., 2014). Both toxin-based and genetic models are suitable for studying these non-motor symptoms that are increasingly recognized as relevant in disease-state (McDowell and Chesselet, 2012). Toxins-based models have been mostly used to seek the mechanisms involved in levodopa induced dyskinesias (LID) thus far (Morin et al., 2014). However, recently viral vector-mediated silencing of TH was used to induce striatal DA depletion without affecting the anatomical integrity of the presynaptic terminals and study LID (Ulusoy et al., 2010). And more recently, for the first time, a genetic mouse model overexpressing A53T α-syn in nigrostriatal and corticostriatal projection neurons shows involuntary movements and increased post-synaptic sensitivity to apomorphine (Brehm et al., 2014). It seems unlikely that a single model can fully recapitulate the complexity of the human disease. Future models should involve a combination of neurotoxin and genetic animal models in order to study the progressive neurodegeneration associated with PD. Understanding the mechanisms responsible for this progressive and intrinsic SNc neuronal loss is completely necessary at this point.

## Conflict of Interest Statement

The authors declare that the research was conducted in the absence of any commercial or financial relationships that could be construed as a potential conflict of interest.
